# Curcumin, a diferuloylmethane, attenuates cyclosporine-induced renal dysfunction and oxidative stress in rat kidneys

**DOI:** 10.1186/1471-2210-5-15

**Published:** 2005-10-15

**Authors:** Naveen Tirkey, Gaganjit Kaur, Garima Vij, Kanwaljit Chopra

**Affiliations:** 1 Pharmacology division, University Institute of Pharmaceutical Sciences, Panjab University, Chandigarh-160014, India

## Abstract

**Background:**

In India, Curcumin (CMN) is popularly known as "Haldi", and has been well studied due to its economic importance. Traditional Indian medicine claims the use of its powder against biliary disorders, anorexia, coryza, cough, diabetic wounds, hepatic disorder, rheumatism and sinusitis. This study was designed to examine the possible beneficial effect of CMN in preventing the acute renal failure and related oxidative stress caused by chronic administration of cyclosporine (CsA) in rats. CMN was administered concurrently with CsA (20 mg/kg/day s.c) for 21 days. Oxidative stress in kidney tissue homogenates was estimated using thiobarbituric acid reactive substances (TBARS), reduced glutathione (GSH) content, superoxide dismutase (SOD), and Catalase (CAT). Nitrite levels were estimated in serum and tissue homogenates.

**Results:**

CsA administration for 21 days produced elevated levels of TBARS and marked depletion of renal endogenous antioxidant enzymes and deteriorated the renal function as assessed by increased serum creatinine, Blood Urea Nitrogen (BUN) and decreased creatinine and urea clearance as compared to vehicle treated rats. CMN markedly reduced elevated levels of TBARS, significantly attenuated renal dysfunction increased the levels of antioxidant enzymes in CsA treated rats and normalized the altered renal morphology.

**Conclusion:**

In conclusion our study showed that CMN through its antioxidant activity effectively salvaged CsA nephrotoxicity.

## Background

Cyclosporine (CsA) (formerly called cyclosporine A), a hydrophobic cyclic undecapeptide produced by the fungus *Tolypocladium inflatum*, can be considered the prototype of immunosuppressant that has revolutionized the management of allotransplantation. This drug specifically and reversibly inhibits immunocompetent T-helper lymphocytes by suppressing the interleukin-2 driven proliferation of activated T-cells [1]. CsA combines low myelotoxicity with effectiveness in preventing allograft rejection and graft versus host disease as well as in the treatment of various autoimmune and ocular inflammatory diseases [2]. Nephrotoxicity and hypertension are the major adverse effects that often limit CsA treatment following solid organ transplantation and autoimmune diseases [3]. The functional changes caused by CsA are dose dependant and are usually reversible after short-term CsA treatment [4].

Cumulative data suggest a role for reactive oxygen metabolites as one of the postulated mechanisms in the pathogenesis of CsA nephrotoxicity. CsA results in enhanced generation of hydrogen peroxide in cultured hepatocytes [5] and mesangial cells [6,7]. In vitro and in vivo studies indicate that CsA enhances lipid peroxidation, reduces renal microsomal NADPH cytochrome P450, and renal reduced/oxidized glutathione ratio (GSH/GSSG) in kidney cortex as well as renal microsomes and mitochondria [8-11]. Antioxidants such as α-tocopherol, ascorbate, silibinin, lazaroid, propionyl carnitine and superoxide dismutase/catalase, have been shown to ameliorate cyclosporine-induced renal toxicity [5,12].

Current traditional Indian medicine claims the use of *Curcuma longa *L. *(Zingiberaceae) *powder against biliary disorders, anorexia, coryza, cough, diabetic wounds, hepatic disorder, rheumatism and sinusitis [13]. Curcumin (CMN) is a major component in curcuma/turmeric, being responsible for its biological actions. More and more studies now show that CMN exhibit anti-inflammatory[14,15], anti-human immunodeficiency virus [16,17], anti-bacterial [18] and nematocidal activities [19]. Various *in-vitro *and *in-vivo *studies increasingly establish the antioxidant properties of CMN [20-22]. It is well documented that CMN scavenges superoxide anions [23], peroxynitrite radicals [24,25], and quenches singlet oxygen [26]. CMN has also been shown to inhibit hydrogen-peroxide-induced cell damage [20].

Thus the present study was designed to examine the possible beneficial effect of CMN in preventing the acute renal failure and related oxidative stress caused by chronic administration of CsA in rats.

## Results

### Effect of CMN on renal function

CsA treatment for 21 days significantly increased the serum creatinine and blood urea nitrogen (BUN) as compared with the control group. Chronic CMN treatment significantly and dose-dependently prevented this rise in BUN and serum creatinine (Table-1). Moreover, the creatinine and urea clearance, which was markedly reduced by CsA-administration, was significantly and dose-dependently improved by CMN treatment (Table-1). However, CMN (15 mg/kg) *per se *had no effect on serum creatinine, BUN, creatinine and urea clearance.

**Table 1:** Effect of CMN on cyclosporine-induced nephrotoxicity

***Variables***	***Control***	***CsA (20)***	***CMN(15)***	***CsA (20)+ CMN(5)***	***CsA (20)+ CMN(10)***	***CsA (20)+ CMN(15)***
Serum creatinine (mg/dl)	0.95 ± 0.01	3.12 ± 0.17^**a**^	0.87 ± 0.01^**b**^	2.00 ± 0.11^**a,b**^	1.5 ± 0.06^**a,b**^	1.00 ± 0.01^**a,b**^
Creatinine clearance (ml/min)	0.76 ± 0.06	0.078 ± 0.05^**a**^	0.87 ± 0.05^**b**^	0.44 ± 0.03^**a,b**^	0.65 ± 0.04^**a,b**^	0.80 ± 0.05^**b**^
BUN (mg/dl)	24.55 ± 0.77	87.44 ± 4.37^**a**^	26.87 ± 0.64^**b**^	73.65 ± 1.32^**a,b**^	53.21 ± 0.9^**a,b**^	35.89 ± 0.64 ^**a,b**^
Urea clearance (ml/min)	0.58 ± 0.04	0.19 ± 0.05^**a**^	0.61 ± 0.03^**b**^	0.49 ± 0.02^**a,b**^	0.53 ± 0.03^**a,b**^	0.59 ± 0.03^**b**^

Values are expressed mean ± mean. a = Statistical significant at P < 0.05 as compared to control, b = Statistical significant at P < 0.05 as compared to Cyclosporine (CsA)

### Effect of CMN on CsA-induced nitrosative stress

Serum and tissue nitrite levels were significantly elevated by CsA-administration. Curcumin treatment significantly and dose dependently improved this increase in nitrite levels both in serum and tissue (Table-2). However, CMN (15 mg/kg) *per se *had no effect on serum nitrite levels.

**Table 2:** Effect of CMN on cyclosporine-induced Nitrite levels

***Variables***	***Control***	***CsA (20)***	***CMN(15)***	***CsA (20)+ CMN(5)***	***CsA (20)+ CMN(10)***	***CsA (20)+ CMN(15)***
Serum Nitrite(μmol/ml)	62 ± 3.72	91.9 ± 50.6^**a**^	60 ± 3.15^**b**^	77 ± 4.55^**a,b**^	69 ± 8.75^**b**^	61 ± 3.05^**b**^
Tissue nitrite(μmol/mg)	103.518 ± 2.73	190.656 ± 7.97^**a**^	101.814 ± 2.27^**b**^	174.704 ± 4.01^**a,b**^	144.79 ± 3.01^**a,b**^	116.912 ± 2.27^**a,b**^

Values are expressed mean ± mean. a = Statistical significant at P < 0.05 as compared to control, b = Statistical significant at P < 0.05 as compared to Cyclosporine (CsA)

### Effect of CMN on CsA-induced lipid peroxidation

**Figure 1:**
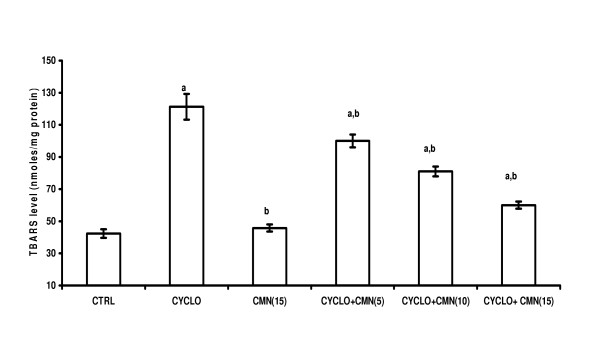
Effect of curcumin (CMN) on Cyclosporine-induced lipid peroxidation in rat kidney. Values are expressed mean ± mean. a = Statistical significant at P < 0.05 as compared to control, b = Statistical significant at P < 0.05 as compared to Cyclosporine (CsA).

Renal TBARS levels were markedly increased by CsA administration as compared to control group. Treatment with curcumin produced a significant and dose-dependent reduction in TBARS in CsA-treated rats, however curcumin per se did not alter TBARS (Fig. 1).

### Effect of CMN on CsA-induced changes in the antioxidant profile

**Figure 2:**
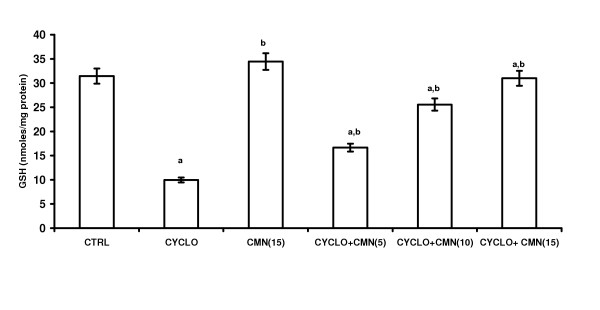
Effect of curcumin (CMN) on Cyclosporine-induced Reduced Glutathione in rat kidney. Values are expressed mean ± mean. a = Statistical significant at P < 0.05 as compared to control, b = Statistical significant at P < 0.05 as compared to Cyclosporine (CsA).

**Figure 3:**
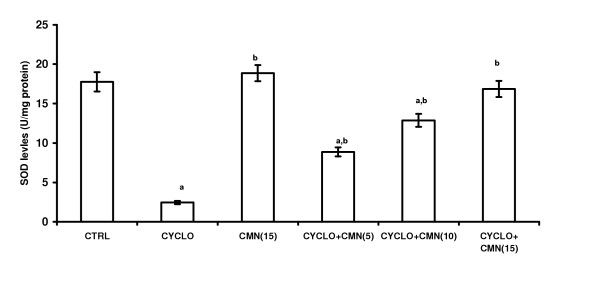
Effect of curcumin (CMN) on Cyclosporine-induced SOD levels in rat kidney. Values are expressed mean ± mean. a = Statistical significant at P < 0.05 as compared to control, b = Statistical significant at P < 0.05 as compared to Cyclosporine (CsA).

**Figure 4:**
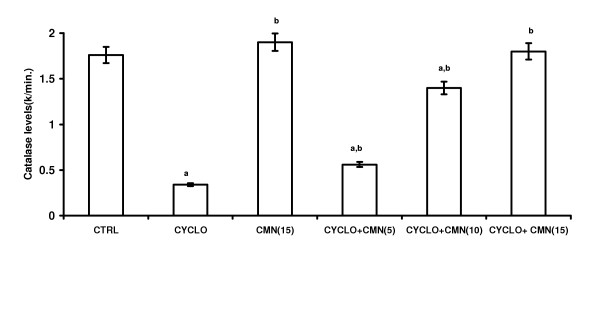
Effect of curcumin (CMN) on Cyclosporine-induced catalase levels in rat kidney. Values are expressed mean ± mean. a = Statistical significant at P < 0.05 as compared to control, b = Statistical significant at P < 0.05 as compared to Cyclosporine (CsA).

Treatment with CsA significantly decreased the reduced glutathione (GSH) levels (Fig. 2) and activities of superoxide dismutase (SOD) (Fig. 3) and catalase (CAT) (Fig. 4). This reduction was significantly and dose-dependetly improved by the treatment with curcumin. However curcumin per se did not alter the endogenous antioxidant profile.

### Effect of CMN on CsA-induced changes on renal morphology

The histopathological changes were graded and summarized in (Table 3). The sections of the control group showed normal glomeruli, afferent arterioles, and tubule cells. By contrast, the kidneys of rats treated with CsA showed marked histological changes in the cortex and outer medulla. The renal sections showed marked tubulointerstital fibrosis, severe epical blebbing and hyaline casts and glomerular basement thickening. Treatment with CMN preserved the normal morphology of the kidney and shows normal glomeruli, no cast formation and slight oedema of the tubular cells.

**Table 3:** Effect of curcumin (15 mg/kg) treatment on morphological changes as assessed by histopathological examination of kidney in Cyclosporine treated rats

**Group**	**Tubular brush border loss**	**Interstitial oedema**	**Tubular dilatation**	**Necrosis of epithelium**	**Hyaline casts**
Control	-	-	-	-	-
CsA	+++	+++	+++	+++	+++
CMN+ CsA	+/-	+/-	+/-	+/-	+/-
CMN	-	-	-	-	-

## Discussion

The exact mechanism of CsA-induced hypertension and nephrotoxicity remain obscure but several studies suggest that a defect in intracellular calcium handling [27], magnesium deficiency [28], oxidative stress [29,30], and nitric oxide (NO) system [31] are involved. Acute renal failure due to CsA is widely attributed to the generation of reactive oxygen species (ROS) by CsA.

It has been reported that binding of pimonidazole, a hypoxia marker in the kidneys, was increased nearly threefold by CsA, indicating marked tissue hypoxia [32]. Moreover, free radicals in the urine were increased dramatically after CsA treatment. [7]. It is also known that CsA increases renal nerve activity resulting in vasoconstricton in the kidney [33]. In addition, CsA causes vasoconstriction directly in isolated renal arterioles [34,35]. It has been demonstrated that CsA blocks mitochondrial Calcium (Ca^+2^) release, inducing a drastic enhancement in intracellular free Ca^+2^, which could account for the vasoconstrictive effect of CsA [36,37]. These alterations could theoretically lead to a classical hypoxia-reoxygenation injury involving oxygen free radicals. In addition, ROS could be derived directly from CsA or during its metabolism by the CYP450 system [6]. It has been demonstrated that cyclosporine increased level of superoxide (O_2_^-^) in endothelial and mesangial cells [9]. Studies show that CsA-induced local production of hydroxyl radical, a highly active and detrimental radical, plays an important role in CsA nephrotoxicity [38].

Couple of studies suggested that CsA induces apoptosis characterized by internucleosomal DNA cleavage due to endonuclease activation, chromatin condensation, and apoptotic bodies in hematopoietic cells [39,40]. Because oxidants are capable of inducing apoptosis in various types of cells [41], including renal tubular epithelial cells [42]. It is conceivable that reactive oxygen metabolites may play a role in apoptotic mechanism of CsA-induced nephrotoxicity.

The present study revealed that chronic administration of CsA for 21 days caused a marked impairment of renal function alongwith significant oxidative stress in the kidneys. Curcumin significantly and dose-dependently improved creatinine and urea clearance, and decreased the elevated levels of serum creatinine and BUN. Earlier studies have also shown that CMN pretreatment decreases ischemia-reperfusion induced rise in serum creatinine levels in kidney [43]. Chronic administration of CsA also produced oxidative stress and increased the lipid peroxidation in kidneys as is seen by the renal TBARS levels. This effect of CsA was again ameliorated by CMN treatment and is in line with various previous reports, which show that CMN decreases lipid peroxidation possibly by its antioxidant mechanism [44]. Oxidative stress can promote the formation of a variety of vasoactive mediators that can affect renal function directly by causing renal vasoconstriction or decreasing the glomerular capillary ultrafiltration coefficient; and thus reducing glomerular filtration rate [45]. Thus the attenuation of lipid peroxidation in CsA-treated rats by CMN provides a convincing evidence for the involvement of ROS in CsA-induced lipid peroxidation. Rukkumani et al. [46] reported protective effect of CMN on circulating lipids in plasma and lipid peroxidation products in alcohol and polyunsaturated fatty acid-induced toxicity. In-vitro findings support the hypothesis that CMN inhibits free radical induced apoptosis in cell lines [47]. Sreejayan et al claimed that the CMN inhibit iron-catalyzed lipid peroxidation in rat brain tissue homogenates by chelation of iron[48].

More and more studies now established the ability of CMN to mainly eliminate the hydroxyl radical [49], superoxide radical [50], singlet oxygen[51], nitrogen dioxide[52] and NO[53]. It has also been demonstrated that CMN inhibits the generation of the superoxide radical[54]. In our study, CsA administration caused marked deterioration of endogenous antioxidant profile as evidenced by decrease in SOD and CAT activities, an effect which was effectively reversed by CMN treatment. Vajragupta et al., [23] have reported that CMN manganese complex and acetylcurcumin manganese complex, low molecular weight synthetic compounds, showed much greater SOD activity and an inhibitory effect on lipid peroxidation. Priyadarsini et al. [55] have shown, by DPPH scavenging in vitro, that origin of the antioxidant activity of CMN is mainly from the phenolic OH group, although a small fraction may be due to the >CH_2 _site.

Further GSH, a major nonprotein thiol in living organisms plays a crucial role in coordinating the body's antioxidant defense processes. Results in the present study indicate that CsA administration drastically lowered the levels of GSH in the kidney. Improvement of renal GSH levels in CMN treated rats in comparison to CsA administered rats further demonstrates the anti-antioxidative effect of CMN. CMN has been shown to increase the levels of glutathione reductase in ischemic brains of rats as well as alveolar and human leukemia cell [20,56,57]. Chronic treatment of CMN also improved the levels of two key antioxidant enzymes SOD and catalase in CsA administered rats.

Peroxynitrite anions have been generated by the reaction of nitric oxide with superoxide anion. These peroxynitrite anions oxidize biomolecules, which finally leads to lipid peroxidation and tubular cell damage [58]. Large amounts of nitric oxide can lead to the depletion of cellular ATP which can inactivate enzymes that contain iron-sulfur clusters, such enzymes involved in mitochondrial electron transport [59]. Nitrosylation of sulfhydryl groups or tyrosine residues in proteins may impair the functional properties of these proteins. Nitric oxide damages DNA, and this in turn, stimulates the DNA repair enzyme poly-ADP-ribose synthetase [60]. Studies done by Amore and colleagues demonstrate that CsA induces apoptosis in various renal cell lines, and this effect is mediated by the induction of iNOS [61]. In line with studies where CMN is reported to inhibit iNOS gene expression in isolated BALB/c mouse peritoneal macrophages and also in the livers of lipopolysaccharide injected mice [62], our study shows that CsA-induced nitrosative stress was significantly and dose dependently attenuated by CMN. Very recently, Sumanont [24] have studied the effect of CMN and its analogues on peroxynitrite anions scavenging activity in vitro using sodium nitroprusside (SNP) generating nitric oxide system. All compounds effectively reduced the generation of NO radicals in a dose dependent manner. They exhibited strong NO radical scavenging activity with low IC(50) values. It is also known that ROS mediates peroxidation of lipid structures of the tissue, resulting in subcellular damage, as observed in histopathological examination. In our study, the kidney of CsA treated rats has shown characteristic morphological findings such as interstial fibrosis and arteriolar hyalinosis. The vasoconstriction induced by CsA produces an ischemic local environment, which leads to a number of cellular changes such as deterioration in membrane integrity the marked histological changes are prominent in the outer cortex and medullary region of the kidney. Because limited oxygen availabity these structures are particularly vulnerable to ischemia. These changes were not observed in the group treated CMN (15 mg/kg) suggesting the protective effect of CMN in attenuating CsA-induced morphological changes.

## Conclusion

In conclusion this study demonstrates that CMN through its marked antioxidant activity coupled with favorable haemodynamic effects salvages CsA nephrotoxicity.

## Methods

### Animals

Wistar albino rats of either sex (150–200 g) were housed in 3 per cage, with food and water ad libitum for several days before the beginning of the experiment. The animals were kept on straw bedding in animal quarters with a natural light: dark cycle. The animals had free access to standard rodent food pellets and water. Animals were acclimatized to the laboratory conditions one day before the start of experiment and daily at least for one hour before the experiment. All the experiments were conducted between 09.00 and 17.00 hrs. The experimental protocols were approved by the Panjab University Animal Ethical Committee.

### Drugs

Curcumin (Sigma Chemicals USA) was suspended in 0.5% Carboxy methyl cellulose (CMC) and administered orally. CsA was a gift from Panacea Biotech India.

### Study design

Rats were divided into six groups each consisting of 5 to 6 animal. Group I received vehicle of CsA i.e. olive oil, subcutaneously (s.c.) and 0.5% Carboxy methyl cellulose (CMC, vehicle for CMN) orally for 21 days. Group II received CsA (20 mg/kg/day, s.c.) dissolved in olive oil for 21 days. This group served as positive control. Three different doses of CMN were tested in Group III, IV, V in which animals received both CsA (20 mg/kg/day s.c) and CMN 5,10,15 mgkg^-1 ^respectively for 21 days. A VI group received only CMN 15 mgkg^-1 ^for 21 days so as to see its *per se *effect. CsA dose was selected from previous studies done in our laboratory. On 21^st ^day of CsA treatment, animals were immediately kept in individual metabolic cages after drug administration for collection of urine. The animals were sacrificed after 24 hr and all the estimations were done as described later.

### Assessment of renal functions

Before sacrifice, rats were kept individually in metabolic cages for 24 h to collect urine for estimation of renal function. A midline abdominal incision was performed and both the kidneys were isolated, the left kidney was deep frozen till further enzymatic analysis, whereas, the right kidney was stored in 10% formalin for the histological studies. Plasma samples were assayed for blood urea nitrogen (BUN), urea clearance, serum creatinine & creatinine clearance by using standard diagnostic kits (Span Diagnostics, Gujarat, India).

### Assessment of oxidative stress

#### Post mitochondrial supernatant preparation (PMS)

Kidneys were, perfused with ice cold saline (0.9% sodium chloride) and homogenized in chilled potassium chloride (1.17%) using a homogenizer. The homogenates were centrifuged at 800 g for 5 minutes at 4°C to separate the nuclear debris. The supernatant so obtained was centrifuged at 10,500 g for 20 minutes at 4°C to get the post mitochondrial supernatant which was used to assay catalase and superoxide dismutase (SOD) activity.

#### Estimation of lipid peroxidation

The malondialdehyde (MDA) content, a measure of lipid peroxidation, was assayed in the form of thiobarbituric acid reacting substances (TBARS) by the method of Okhawa et al. [63]. Briefly, the reaction mixture consisted of 0.2 ml of 8.1% sodium lauryl sulphate, 1.5 ml of 20% acetic acid solution adjusted to pH 3.5 with sodium hydroxide and 1.5 ml of 0.8% aqueous solution of thiobarbituric acid was added to 0.2 ml of 10%(w/v) of PMS. The mixture was brought up to 4.0 ml with distilled water and heated at 95°C for 60 minutes. After cooling with tap water, 1.0 ml distilled water and 5.0 ml of the mixture of n-butanol & pyridine (15:1 v/v) was added and centrifuged. The organic layer was taken out and its absorbance was measured at 532 nm. TBARS were quantified using an extinction coefficient of 1.56 × 10^5 ^M^-1^/cm^-1 ^and expressed as nmol of TBARS per mg protein. Tissue protein was estimated using Biuret method of protein assay and the renal MDA content expressed as nanomoles of malondialdehyde per milligram of protein.

#### Estimation of reduced glutathione

Reduced glutathione (GSH) in the kidneys was assayed by the method of Jollow et al [64]. Briefly, 1.0 ml of PMS (10%) was precipitated with 1.0 ml of sulphosalicylic acid (4%). The samples were kept at 4°C for at least 1 hour and then subjected to centrifugation at 1200 g for 15 minutes at 4°C. The assay mixture contained 0.1 ml filtered aliquot and 2.7 ml phosphate buffer (0.1 M, pH 7.4) in a total volume of 3.0 ml. The yellow colour developed was read immediately at 412 nm on a spectrophotometer.

#### Estimation of superoxide desmutase(SOD)

SOD activity was assayed by the method of Kono et al[65] The assay system consisted of EDTA 0.1 mM, sodium carbonate 50 mM and 96 mM of nitro blue tetrazolium (NBT). In the cuvette, 2 ml of above mixture, 0.05 ml hydroxylamine and 0.05 ml of PMS were taken and the auto-oxidation of hydroxylamine was observed by measuring the absorbance at 560 nm.

#### Estimation of catalase

Catalase activity was assayed by the method of Claiborne et al [66]. Briefly, the assay mixture consisted of 1.95 ml phosphate buffer (0.05 M, pH 7.0), 1.0 ml hydrogen peroxide (0.019 M) and 0.05 ml PMS (10%) in a final volume of 3.0 ml. Changes in absorbance were recorded at 240 nm. Catalase activity was calculated in terms of k minutes-1.

### Assessment of serum/tissue nitrite concentration

Serum and tissue nitrite was estimated using Greiss reagent and served as an indicator of NO production. 500 μl of Greiss reagent (1:1 solution of 1% sulphanilamide in 5% phosphoric acid and 0.1% napthaylamine diamine dihydrochloric acid in water) was added to suitably diluted 100 μl of plasma and absorbance was measured at 546 nm [67]. Nitrite concentration was calculated using a standard curve for sodium nitrite. Nitrite levels were expressed as μmol/ml in serum and as μmol/mg protein in homogenate.

### Histopathological examination

For microscopic evaluation kidney were fixed in 10% neutral phosphatebuffered formalin solution. Following dehydration in ascending series of ethanol (70, 80, 96, 100%), tissue samples were cleared in xylene and embedded in paraffin. Tissue sections of 5 μm were stained with hematoxylin-eosin (H-E). A minimum of 10 fields for each kidney slide were examined and assigned for severity of changes by an observer blinded to the treatments of the animals and assigned for severity of changes using Scores of none (-), mild (+), Moderate (++) and Severe (+++)

### Statistical analysis

Results were expressed as mean± SEM. The intergroup variation was measured by one way analysis of variance (ANOVA) followed by Fischer's LSD test. Statistical significance was considered at p < 0.05. The statistical analysis was done using the Jandel Sigma Stat Statistical Software version 2.0.

## Authors' contributions

Naveen Tirkey, Gangandeep kaur and Garima Vij did all the biochemical estimations in kidney and did the data interpretation after statistical analysis. Kanwaljit Chopra contributed in manuscript preparation.

**Figure 5:**
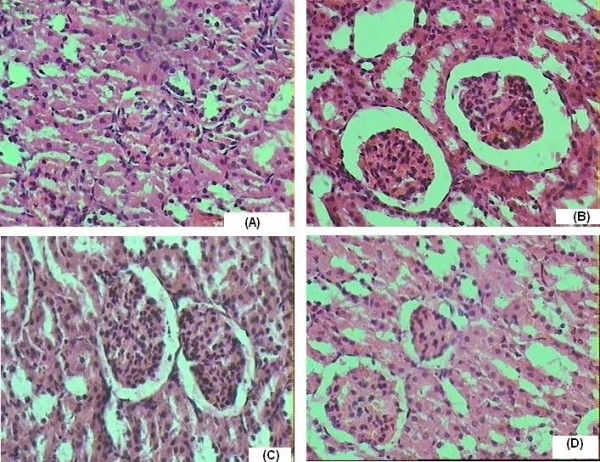
(A) Hematoxylin and Eosin-stained sections of Normal rat kidneys. (B) Kidney section of CsA treated rats showing tubular brush-border loss, interstitial oedema, Necrosis of epithelium and Hyaline Casts. (C) Kidney Section of CMN (15 mg/kg p.o) + CsA treated rats showing prevention of CsA induced alterations. (D) Kidney section of CMN (15 mg/kg p.o.) treated rats showing almost normal morphology.
